# Epithelial Haven and Autophagy Breakout in Gonococci Infection

**DOI:** 10.3389/fcell.2020.00439

**Published:** 2020-06-09

**Authors:** Ana Clara Mendes, Marcone Ciccone, Bruna Gazolla, Diana Bahia

**Affiliations:** Departamento de Genética, Ecologia e Evolução, Instituto de Ciencias Biológicas, Universidade Federal de Minas Gerais, Belo Horizonte, Brazil

**Keywords:** *N. gonorrhoeae*, autophagy, epithelial cell, intracellular pathogen, epithelial cell invasion

## Abstract

The World Health Organization (WHO) has estimated that in 2016, there were 87 million new cases of gonorrhea. Gonorrhea is caused by the sexually transmitted human-exclusive agent *Neisseria gonorrhoeae*, a Gram-negative diplococcus that causes cervicitis in females and urethritis in males and may lead to more severe complications. Currently, there is no vaccine against *N. gonorrhoeae*. Its resistance to antibiotics has been increasing in the past few years, reducing the range of treatment options. *N. gonorrhoeae* requires a surface protein/receptor (Opa proteins, porin, Type IV pili, LOS) to adhere to and invade epithelial cells. During invasion and transcytosis, *N. gonorrhoeae* is targeted by the autophagy pathway, a cellular maintenance process which balances sources of energy at critical times by degrading damaged organelles and macromolecules in the lysosome. Autophagy is an important host defense mechanism which targets invading pathogens. Based on transmission electron microscopy (TEM) analysis, the intracellular bacteria occupy the autophagosome, a double-membraned vesicle that is formed around molecules or microorganisms during macroautophagy and fuses with lysosomes for degradation. Most of the gonococci end up in autolysosomes for degradation, but a subpopulation of the intracellular bacteria inhibits the maturation of the autophagosome and its fusion with lysosomes by activating mTORC1 (a known suppressor of the autophagy signaling), thus escaping autophagic elimination. This mini review focuses on the cellular features of *N. gonorrhoeae* during epithelial cell invasion, with a particular focus on how *N. gonorrhoeae* evades the autophagy pathway.

## Introduction

*Neisseria gonorrhoeae*, also known as gonococcus, is the causative agent of gonorrhea, a sexually transmitted infection that occurs exclusively in humans. In 2016, The World Health Organization (WHO) estimated that 86.9 million new cases of gonorrhea occurred globally. In 2017, the WHO included *N. gonorrhoeae* in its list of bacteria for which new antibiotics are urgently needed^1^. *N. gonorrhoeae* is a major global public health concern due to its increasing resistance to antibiotics, which leads to the possibility of untreatable gonorrhea infections (World Health Organization [WHO], 2017; Rowley et al., 2019). *N. gonorrhoeae* is a Gram-negative diplococcus that usually infects urogenital epithelia, but it is also able to infect rectal, pharynx, and conjunctival mucosa (Britigan et al., 1985).

At the sites of gonococci colonization, the activation of the innate immune response causes the symptoms of gonorrhea, including discomfort in the affected area and purulent urethral or cervical discharge. Acute gonorrhea results in an intensely inflammatory exudate, which contains macrophages, exfoliated epithelial cells, and polymorphonuclear neutrophils (Hook, 2012). Many studies have shown that asymptomatic infections are common in both men and women, but are more prevalent in women than in men (Muzny et al., 2017). This may be due to the relative ease in diagnosing symptoms in men, as the purulent exudate causes painful urination in men. Symptoms in women are mostly unnoticed and/or non-specific and are often mistaken for symptoms of bacterial vaginosis, hormonal alterations, or normal vaginal secretions (Grimley et al., 2006; Quillin and Seifert, 2018). Untreated gonorrhea may result in pelvic inflammatory disease, infertility, ectopic pregnancies, or neonatal blindness as a consequence of vertical transmission. In addition, untreated gonorrhea can lead to gonococcal dissemination and enhanced transmission of HIV (Masi and Eisenstein, 1981; Sandstrom, 1987; Little, 2006).

## Adherence and Invasion

*Neisseria gonorrhoeae* adheres to urogenital tract by attaching to surface structures as Type IV pili (Tfp) (Pearce and Buchanan, 1978), opacity (Opa) proteins, LOS, or outer membrane protein porin (PorB) (Stern et al., 1986; van Putten and Paul, 1995). Type IV pili (Tfp) mediate initial cellular adherence, its retraction brings the bacteria closer to the epithelial cell surface and activates Ca^2+^ flux, PI3K/Akt, and the ERK/MAP kinase pathways (Ayala et al., 2005; Lee et al., 2005). The Opa family of proteins includes two classes: the Opa50 protein, which binds to surface heparan sulfate proteoglycan (HSPG) receptors; and Opa51-60, which bind to carcinoembryonic antigen-related cellular adhesion molecules (CEACAMs) and mediate the complex interactions between the gonococci and epithelial cells or phagocytes after Tfp adhesion (van Putten and Paul, 1995). After adhesion, *N. gonorrhoeae* replicates in microcolonies, which are collections of bacteria formed from a few diplococci after the initial adhesion on epithelial cells, competes with the local microbiota, and is able to invade and disseminate by transmigrating across the epithelial cell monolayer (Quillin and Seifert, 2018). Gonococcal microcolonies can move and promote interaction between bacterial cells, helping them to deal with environmental pressures. In addition, microcolonies play a role in gonococci-host interactions (Higashi et al., 2007).

The gonococci initiate cross-talk with host cells using multiple surface molecules, resulting in activation of signaling pathways and changes in gene expression in the host cells and in the gonococci themselves (Stein et al., 2015). Interactions between CEACAMs and Opa proteins can induce phagocytosis, triggering the engulfment of the bacteria into the epithelial cells and neutrophils (Fox et al., 2014).

*Neisseria gonorrhoeae* facilitates its invasion into host cells by modulating the activity and distribution of host epidermal growth factor receptor (EGFR), which is a signaling receptor that pathogens can manipulate for their survival in host cells (Zhang et al., 2004). The gonococcal microcolonies recruit EGFR to the site of microcolony adherence. The kinase activity of EGFR is necessary for gonococcal invasion into epithelial cells. The gonococci activate EGFR by inducing the production of EGFR ligands. This suggests that microcolonies are important for invasion of *N. gonorrhoeae* into epithelial cells. Studies have shown that EGFR kinase inhibition reduces gonococcal invasion, further indicating an important role for EGFR in gonococci invasion (Swanson et al., 2011).

Apicella et al. (1996) analyzed urethral exudates from men infected with *N. gonorrhoeae* and observed that bacteria were clustered within vacuoles upon invasion. However, they also found bacteria in the cytosol without evidence of a surrounding vacuole. Harvey et al. (1997) studied primary human urethral epithelial cell cultures infected with *N. gonorrhoeae* and showed that after the invasion, the gonococci appeared to reside and multiply within vacuoles close to the apical layers of the epithelial cells and were later released from the epithelial cell monolayer either in membrane-bound vacuoles or after rupturing the infected cells (Mosleh et al., 1997). Gonococcal infection of the urethral epithelium modulates host anti-apoptotic factors, thereby promoting bacterial survival within the epithelial tissue (Binnicker et al., 2003).

## What Is Autophagy?

Autophagy is a cellular mechanism that can be upregulated in response to stress conditions and lack of nutrients. It is a pathway that delivers organelles and cytoplasmic proteins to the lysosome for degradation (Yang et al., 2005).

Mammalian cells have three types of autophagy: microautophagy, in which the lysosome captures the molecules by invagination of the lysosomal membrane; chaperone-mediated autophagy (CMA), in which chaperone proteins identify molecules that contain a particular pentapeptide motif and transport them directly to lysosomes; and macroautophagy, referred to in this text as autophagy, which involves the formation of cytosolic vesicles to transport the molecules, including damaged organelles or pathogens, to the lysosome for degradation. Upon activation of autophagy, a membrane structure known as a phagophore forms and expands, eventually closing to form a double-membrane vesicle called autophagosome (Parzych and Klionsky, 2014).

Autophagosomes fuse with lysosomes (autolysosomes), and the sequestered cargo is digested. The initial formation of the autophagosomes requires the activation of the unc-51-like kinase 1 (ULK1) complex. Then, ULK activates the Vps34 (class III phosphatidylinositol 3-kinase) complex, which comprises Vps34, associated to Beclin 1, VPS15, and ATG14L, triggering vesicle nucleation. The subsequent steps involve the ATG12 and the LC3 (microtubule-associated light chain 3) conjugation systems. Both systems promote the elongation of the isolation membrane (Joubert et al., 2009; Rubinsztein et al., 2012).

An important regulator of autophagy is the target of rapamycin (TOR), which inhibits multiple autophagy-promoting proteins via phosphorylation. TOR is a phosphatidylinositol-related kinase involved in regulatory pathways that control the responses to nutrients and energy metabolism changes. In mammalian cells, mTOR nucleates two structurally and functionally different complexes termed mTORC2, which regulates cytoskeleton organization and cell survival, and mTORC1, which is essential to sense and respond to intracellular and extracellular nutrients, amino acids, growth factors, energy, and oxygen levels. In the presence of stimuli, mTORC1 phosphorylates the ULK1 complex, inhibiting autophagy. On the contrary, when mTORC1 is inactive, ULK1 is released and autophagy is initiated (Yang et al., 2005; Huang and Brumell, 2014; Rabanal-Ruiz et al., 2017).

Although autophagy can be induced to control infection upon intracellular pathogen invasion, many pathogens have developed strategies to avoid or subvert autophagy for their own benefit. Bacteria are targets of selective autophagy, a process known as xenophagy. Xenophagy is a mechanism that targets and removes pathogens after cellular invasion (Bauckman et al., 2015; Escoll et al., 2016). It can be induced upon bacterial infection and involves the formation of autophagosomes, which target bacteria and transport them to lysosomes for degradation. Some bacteria can inhibit autophagy signaling pathways, avoid autophagy recognition, inhibit fusion of the autophagosome with the lysosome, or escape targeting by interfering with the autophagy machinery (Gomes and Dikic, 2014; Bauckman et al., 2015).

CD46 acts as an immunomodulatory protein and plays a role in autophagy signaling. CD46 is a glycoprotein expressed on the surface of every nucleated human cell, and it has isoforms that contain one of two short cytoplasmic tails (cyt), cyt-1 or cyt-2, the most abundant CD46 isoform (Meiffren et al., 2010). CD46 is a cellular receptor for several pathogens, including measles virus, human herpes virus 6, adenovírus B and D, group A *Streptococcus* (GAS), and *Neisseria* bacteria (Cattaneo, 2004).

Joubert et al. (2009) demonstrated that CD46 is connected to autophagy. They found an interaction between the scaffold protein GOPC and cyt-1. GOPC contains two coiled-coil domains (CC) and a PDZ domain, and it interacts with cyt-1 through the PDZ domain. GOPC is reported to interact with Beclin-1, (an important molecule in autophagy induction, part of the Vps34 complex) through CC domains. The CD46-cyt-1/GOPC interaction is associated with the autophagosome formation complex Vps34/Beclin-1, recruiting this complex to initiate autophagy (Joubert et al., 2009).

## Autophagy Induction and Escape

Very little is known about *N. gonorrhoeae*’s interaction with autophagy and its impact on intracellular survival, and recent studies have demonstrated that autophagy does affect the survival of intracellular gonococci. As a consequence of cellular invasion, *N. gonorrhoeae* is targeted by the autophagy pathway: *N. gonorrhoeae* was found within double-membrane autophagic structures by transmission electron microscopy (TEM), suggesting that the gonococcus ended up in autophagosomes (Lu et al., 2019).

**FIGURE 1:**
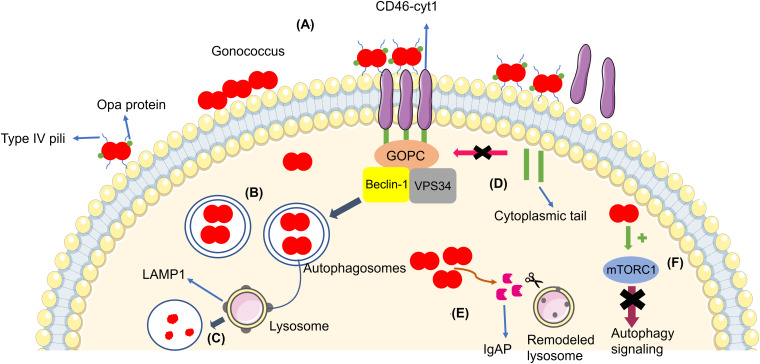
**(A)** Gonococci (in red) adhere to the cellular membrane and form a microcolony, triggering the autophagy pathway through CD46-cyt1/GOPC, which recruits the Beclin-1/Vps34 complex. **(B)** Bacteria become enveloped in the autophagosome (blue arrow). **(C)** The autophagosome fuses with the lysosome in order to kill the bacteria. **(D)** Gonococci evade the autophagy pathway by inducing the cleavage of the CD46-cyt ectodomain via metalloproteinases and by inducing the cleavage and release of the cytoplasmic tail via presenilin/y-secretase, decreasing intracellular CD46-cyt1 and the cell’s ability to initiate autophagy (pink arrow). **(E)** Bacteria secrete IgAP, (orange arrow) which cleaves LAMP1, resulting in remodeling of the lysosomal membranes and prevention of autophagosome/lysosome fusion, therefore increasing the survival of the gonococci. **(F)** Gonococci activate mTORC1 (green arrow), therefore suppressing autophagy signaling (purple arrow).

Kim et al. (2019) reported that *N. gonorrhoeae* (MS11 strain and only piliated and Opa non-expressing bacteria) infection led to autophagosome formation and activation of autophagy in the endocervical cell lines ME180 and Hec1B, induced through CD46-cyt1/GOPC in host cells. The gonococcus interacts with CD46-cyt1 via the Type IV pilus (Tfp), recruiting CD46-cyt1 at the site of infection (Figure 1). Thus, *N. gonorrhoeae* stimulates matrix metalloproteinases, which are host extracellular sheddases that cleave the CD46-cyt1 ectodomain. After the cleavage of the CD46-cyt1 ectodomain, the presenilin/γ-secretase complex, a host membrane protease complex that modifies type I transmembrane protein function and signaling, cleaves the transmembrane domain, resulting in the release of the cytoplasmic domain. Consequently, this complex gradually reduces the pool of intracellular CD46-cyt1, which decreases the ability of infected cells to initiate autophagy (Weyand et al., 2010).

Autophagosome maturation and fusion with the lysosome is inhibited by *N. gonorrhoeae*. Studies have shown that *Legionella pneumophila* (Choy et al., 2012; Arasaki and Tagaya, 2017) and *Mycobacterium tuberculosis* (Vergne et al., 2005) are also able to inhibit autophagosome maturation. Additionally, *N. gonorrhoeae* secrets IgAP, a protein which cleaves the major lysosomal membrane protein LAMP1. IgAP cleaves LAMP1 gradually, remodels lysosomes, and blocks lysosome/autophagosome fusion. This dual interference in the autophagy pathway promotes the survival of *N. gonorrhoeae* until the later stages of infection (Kim et al., 2019).

Lu et al. (2019) quantified intracellular and extracellular bacteria (American Type Culture Collection 49226) in order to evaluate the escape of gonococci from autophagy in HeLa cells. After initial gonococci invasion, the authors added gentamicin in the culture medium to eliminate extracellular bacteria, showing that the extracellular bacteria in the later stages of infection are the subpopulation of the intracellular *N. gonorrhoeae* that survived autophagy degradation and underwent exocytosis, the process through bacteria are transported inside vesicles to extracellular environment. When *N*. *gonorrhoeae* invades cells, many gonococci end up in autophagosomes for elimination, showing that the autophagy pathway affects gonococcus survival in the early stages of invasion. However, a subpopulation of bacteria evades the autophagy pathway (Lu et al., 2019). In the same study, they found that *N. gonorrhoeae* also activates the autophagy repressor mTORC1 during the intracellular stages, resulting in suppression of autophagy signaling and thus subverting autophagy for its own benefit (Figure 1). This strategy is also used by other intracellular pathogens, including *Legionella* sp., *Shigella flexneri*, and *S*almonella *typhimurium* (Ogawa et al., 2005; Choy et al., 2012; Tattoli et al., 2012). In addition, *N. gonorrhoeae* also inhibited autophagosome maturation and autophagolysosome formation in a way independent of mTORC1 activation through a mechanism that remains unclear (Lu et al., 2019).

In order to grow inside epithelial cells, *N*. *gonorrhoeae* uses host sources to acquire iron, resulting in interruption of iron homeostasis in the cells and liberation of bioavailable iron from ferritin storage compartments. This supports the intracellular growth of *N. gonorrhoeae* in the late stages of infection, since autophagy mediates ferritin degradation. Therefore, the autophagic flux in the early stages of invasion may increase iron availability in the cell, resulting in the growth of intracellular *N. gonorrhoeae* in the later stages of infection (Bonnah et al., 2000; Larson et al., 2004; Dowdle et al., 2014).

Transcytosis is the transit across cellular epithelium monolayers into the subepithelial space by a bacterium and usually requires endocytic recycling and vesicular transport systems. The epithelial invasion of *N. gonorrhoeae* and its transcytosis is related to disseminated gonococcal infection and results in complications. The importance of epithelial cell invasion and transcytosis in uncomplicated infections is not clear and needs further investigation (Quillin and Seifert, 2018).

## Conclusion

*Neisseria gonorrhoeae* is already resistant to most antibiotics, which makes the treatment of this disease difficult. In addition, the lack of immunologic memory due to surface antigenic variation complicates the development of an efficient vaccine. Consequently, new studies related to the survival, proliferation, and permanence strategies used by *N. gonorrhoeae* are important. However, studies on the pathogenesis of the bacteria are challenging because *N. gonorrhoeae* is an exclusive human pathogen, which means that it needs specific human proteins to interact and nutrients to grow. As a consequence, animal or culture models that mimic human tissue are needed to better study the pathogenesis of *N. gonorrhoeae*. Some studies have developed such models, although they still have limitations and may not represent all human conditions.

The recent studies of autophagy and *N. gonorrhoeae* infection show that in the early stages of invasion, bacteria survival is impaired by the autophagy pathway. However, in the later stages of infection, some gonococci are capable of subverting autophagy signaling and maintaining the infection. Consequently, targeting specific bacterial proteins related to autophagy inhibition could be another strategy to control the infection. The development of drugs that affect the bacterial-host interactions and not only the bacteria itself would be promising given that it would allow the host innate immune system to respond to the infection upon autophagy reactivation. We can propose, for instance, the development of drug or antibody to antagonize IgAP, which would prevent the blockage of lysosome/autophagosome fusion. Nonetheless, more studies are needed to better understand the interactions between *N. gonorrhoeae* and host autophagy.

## Author Contributions

AM, MC, BG, and DB conceived and wrote the manuscript.

## Conflict of Interest

The authors declare that the research was conducted in the absence of any commercial or financial relationships that could be construed as a potential conflict of interest.

## Footnotes

1 https://www.who.int/medicines/news/bacteria-antibiotics-needed/en/
